# Results of treatment of congenital vertical talus by the Dobbs method

**DOI:** 10.1186/s13018-023-03708-6

**Published:** 2023-04-18

**Authors:** Andrzej Grzegorzewski, Łukasz Lipiński, Błażej Pruszczyński, Paweł Grzegorzewski, Piotr Buchcic

**Affiliations:** Orthopedics and Pediatric Orthopedics Clinic, Medical University of Lodz, Lodz, Poland

**Keywords:** Talus, Congenital, Foot, Dobb’s, Forefoot, Verticalis

## Abstract

Congenital vertical talus is a rare foot deformity. The hindfoot is valgus and equinus, the midfoot is dorsiflexed and forefoot is abducted due to a fixed dorsal dislocation of the navicular on the head of the talus and the cuboid on the anterior part of the calcaneus. The epidemiology and etiology of vertical talus is unknown. Dobbs et al. (J Bone Joint Surg Am 88(6):1192–200, 2006) described a minimally invasive alternative which allowed to avoid the need for extensive soft tissue release procedures in treatment of congenital vertical talus. Eleven congenital vertical talus feet (group 5 according to Hamanishi) in eight children (four boys and four girls) constituted the study material. Upon the diagnosis, the patients’ age ranged from 5 to 26 months old (the mean – 14.6). The treatment involved serial manipulation and casting according to the reverse Ponseti method (from 4 to 7 casts) followed by a minimally invasive approach consisting in temporary stabilization of the talonavicular joint with the use of K-wire and Achilles tenotomy according to the Dobbs technique. Then patients continued the shoe and bar program for 2 years. The X-ray measurements on lateral radiographic included the talocalcaneal angle, tibiotalar angle and talar axis—first metatarsal base angle whereas AP radiographic images—the talocalcaneal angle and talar axis—first metatarsal angle. The Wilcoxon test was used to compare dependent variables. The final clinical assessment made during the last follow-up (the mean: 35.8 months, the range: 25–52) revealed that neutral position of the foot and normal range of motion were observed in ten cases and recurrence of foot deformity in one case. The last X-ray examination showed normalization all of radiological parameters, except for one case, and examined parameters were statistically significant. The minimally invasive technique described by Dobbs should be the first option in treatment of congenital vertical talus. It allows to reduce the talonavicular joint, brings good results and preserves foot mobility. The attention should be put on early diagnosis.

## Introduction

Congenital vertical talus is a rare foot deformity. The hindfoot is valgus and equinus, the midfoot is dorsiflexed and forefoot is abducted due to a fixed dorsal dislocation of the navicular on the head of the talus and the cuboid on the anterior part of the calcaneus. The condition usually misdiagnosed during first weeks or months of newborn life, which is contributed to difficulty in differentiating between the foot abnormality and other more common positional foot anomalies, such as calcaneovalgus deformity, oblique talus or flexible flatfoot [1, 2]. An X-ray of foot is necessary so as to set a proper diagnosis which can be a challenge in early life. The epidemiology and etiology of vertical talus is unknown, the estimated prevalence is 1 in 10,000 live births [3]. A typical surgical management for vertical talus used to be intensive and long. The posterolateral approach which allowed to extend the correction with medial access (one-stage or two-stage approach) or Cincinnati surgical approach were used to restore a normal, anatomical foot. It could lead to a complication including both undercorrection and overcorrection of the deformity, stiffness caused by extensive soft tissue release [4, 5]. Finally, early degenerative joint changes in the foot bones were observed. Dobbs et al., in 2006, described a minimally invasive alternative which allowed to avoid performing extensive soft tissue release procedures in treatment of congenital vertical talus [1].

The aim of this study is to present clinical and radiological results by using a minimally invasive procedures in treatment of congenital vertical talus.

## Methods

Eleven congenital vertical talus feet in eight children (four boys and four girls) constituted the study material. The children did not demonstrate any other health condition, such as: neural tube defects, neuromuscular disorders, malformation syndromes and chromosomal aberrations—group 5 according to Hamanishi [6]. Children were treated consistently by senior author with the application of the Dobbs method. The etiology of foot deformity did not change to the end of the observation. What is worth stressing is the fact that we did not observe generalized joint laxity. In one case we simultaneously observed bilateral vertical talus and bilateral DDH (type IIIa according to the Graff classification), which was successfully treated with a Pavlik harness. Upon the diagnosis, the patients’ age ranged from 5 to 26 months old (the mean: 14.6). A clinical examination revealed a hindfoot equinus and valgus, forefoot dorsiflexion and abduction in all cases. A convex plantar surface (rocker-bottom) was not so clearly visible because of thick fat pad which covered the foot, particularly in older babies. The deformity was rigid with a certain degree of flexibility between the navicular and talar head with plantar flexion of the forefoot. A radiographic evaluation includes weight-bearing (a mimic weight-bearing in younger patients) AP, a lateral view of the foot and a lateral view of the foot in the maximum plantar flexion. Lateral radiographic measurements include the talocalcaneal angle, tibiotalar angle and talar axis—first metatarsal base angle (TAMBA) and on AP radiographs show—the talocalcaneal angle and talar axis—first metatarsal angle (Table 1) [6].

**Table 1:** Mean radiological measurements preoperatively and at last follow-up (all angles shown as range, mean ± standard deviation, 11 feet)

Measurement	Pre-op	Last follow-up	*p* value
AP talocalcaneal	50–70 (55.6 ± 6.44)	15–45 (26.4 ± 8.32)	*p* = 0.001
Lateral talocalcaneal	50–75 (54.4 ± 7.95)	23–59 (32.55 ± 12.06)	*p* = 0.002
AP talar-first metatarsal	39–89 (60.55 ± 17.99)	5–30 (18.64 ± 10.26)	*p* = 0.001
Lateral talar-basis first metatarsal	46–88 (61.82 ± 15.32)	2–45 (13.64 ± 15.85)	*p* = 0.002
Lateral tibiotalar	9–30 (19.55 ± 7.03)	40–75 (66.27 ± 7.07)	*p* = 0.001

Treatment involved an application of serial manipulation and casting followed by a minimally invasive approach with temporary stabilization of the talonavicular joint with K-wire and Achilles tenotomy according to the Dobbs technique. Manipulations involved stretching of the foot into plantar flexion and adduction with simultaneously counter pressure on the tali head, which pushed the talus dorsally and laterally. Then, a plaster cast was applied according to the reverse Ponseti method for one week. Over the entire period, the patient was applied from 4 to 7 casts (the mean: 5.6) in order to, to achieve close reduction in the talonavicular joint before the fixation was attempted [1, 2]. A surgical procedure was made according to the Dobbs technique—the skin was incised medially over the talonavicular joint with joint capsulotomy. K-wire was placed in a retrograde fashion under the C-arm guidance and percutaneous Achilles tenotomy was performed. It was unnecessary to lengthen the peroneus brevis, tibialis anterior and/or dorsal extensor tendons. A long leg cast was applied to the foot in the neutral position for 6 weeks; then K-wire was removed. The total time required for cast treatment was approximately 10–13 weeks (before and after the surgery, the mean: 11.6 weeks) depending on the number of cast changes. No complication were noted during treatment. Then the patient started to use the shoes and bar system (Denis–Brown, 10° of plantar flexion and adduction at the mid-tarsal joint) nonstop for 3–4 months and after this period, he/she wore it only at night time for 2 years. The parents were instructed to regularly stretch dorsolateral soft tissues of the foot 4–6 times a day. The follow-up was 2 years minimum, when the patient discontinued the shoe and bar program.

The study was approved by the Ethical Committee of the Medical University of Lodz.

### Statistical analysis

We used the Wilcoxon test to compare dependent variables (appropriate angular measurements on the same feet before the surgery and at the last follow-up). All statistical analyses were significant for *p* < 0.05 and they were performed using SPSS ver. 24 software (IBM Corp., Armonk, NY).

## Results

**Fig. 1:**
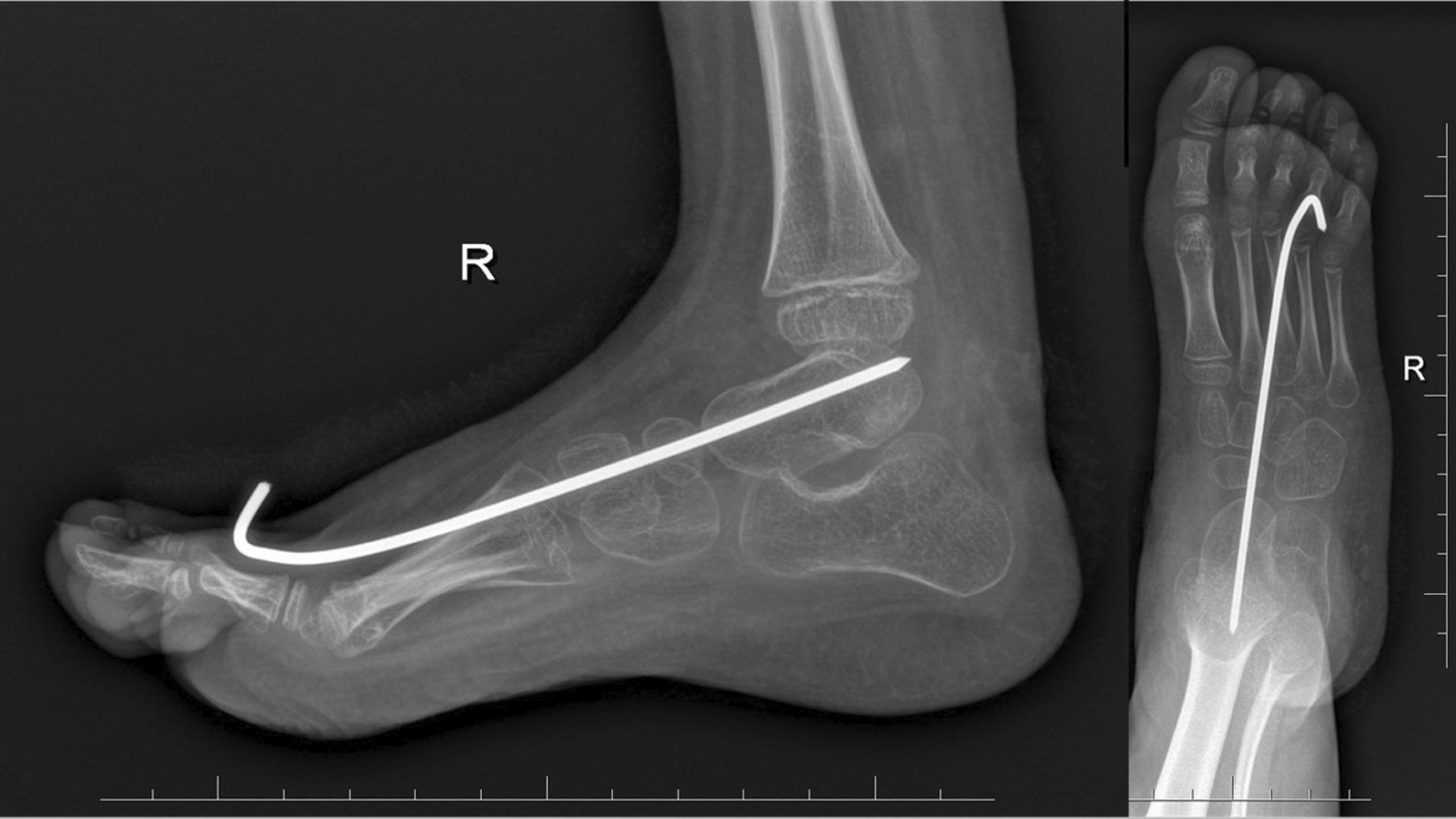
Case 2 AP and L X-ray after surgery

**Fig. 2:**
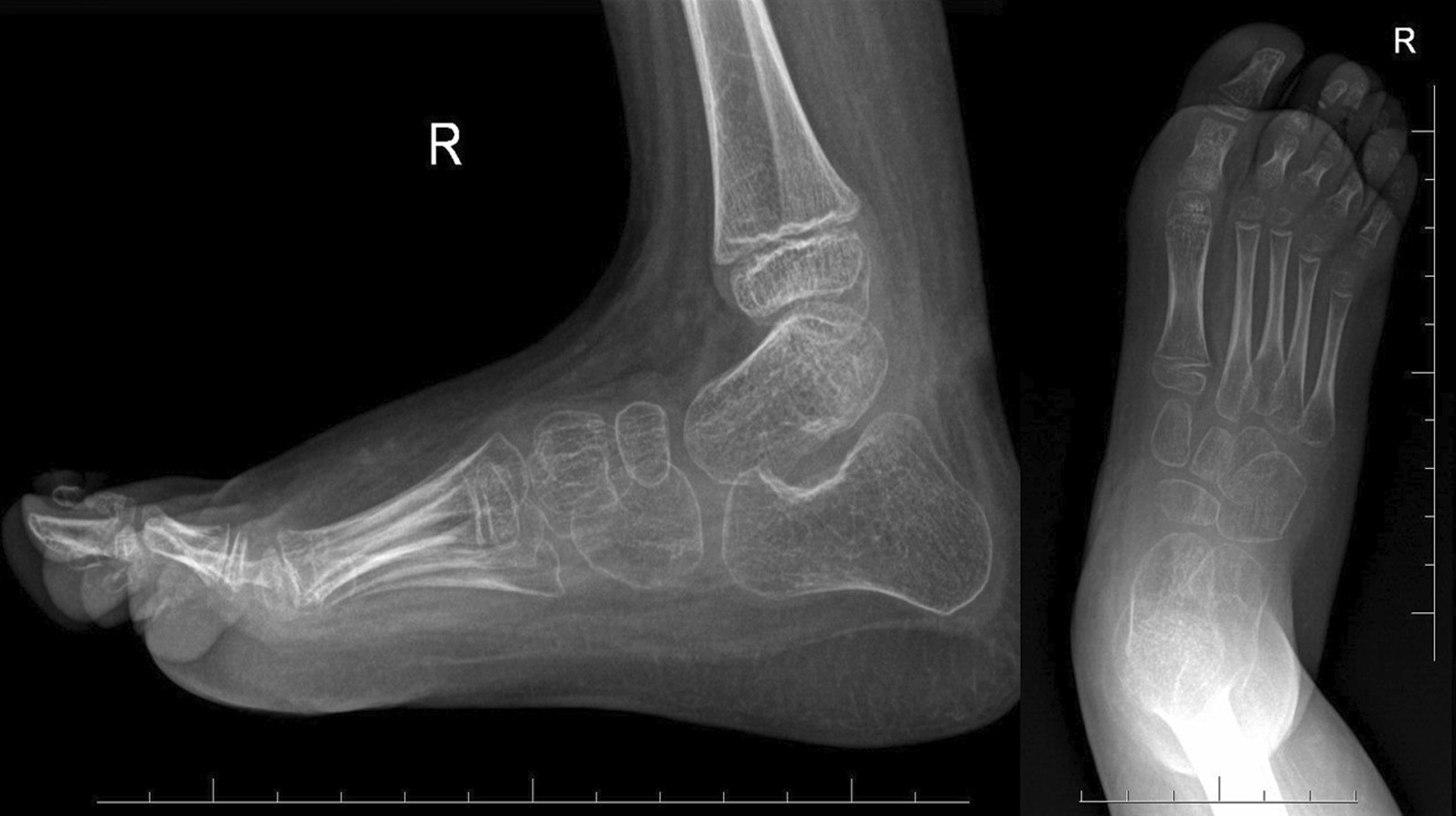
Case 2 AP and L at last follow-up

**Fig. 3:**
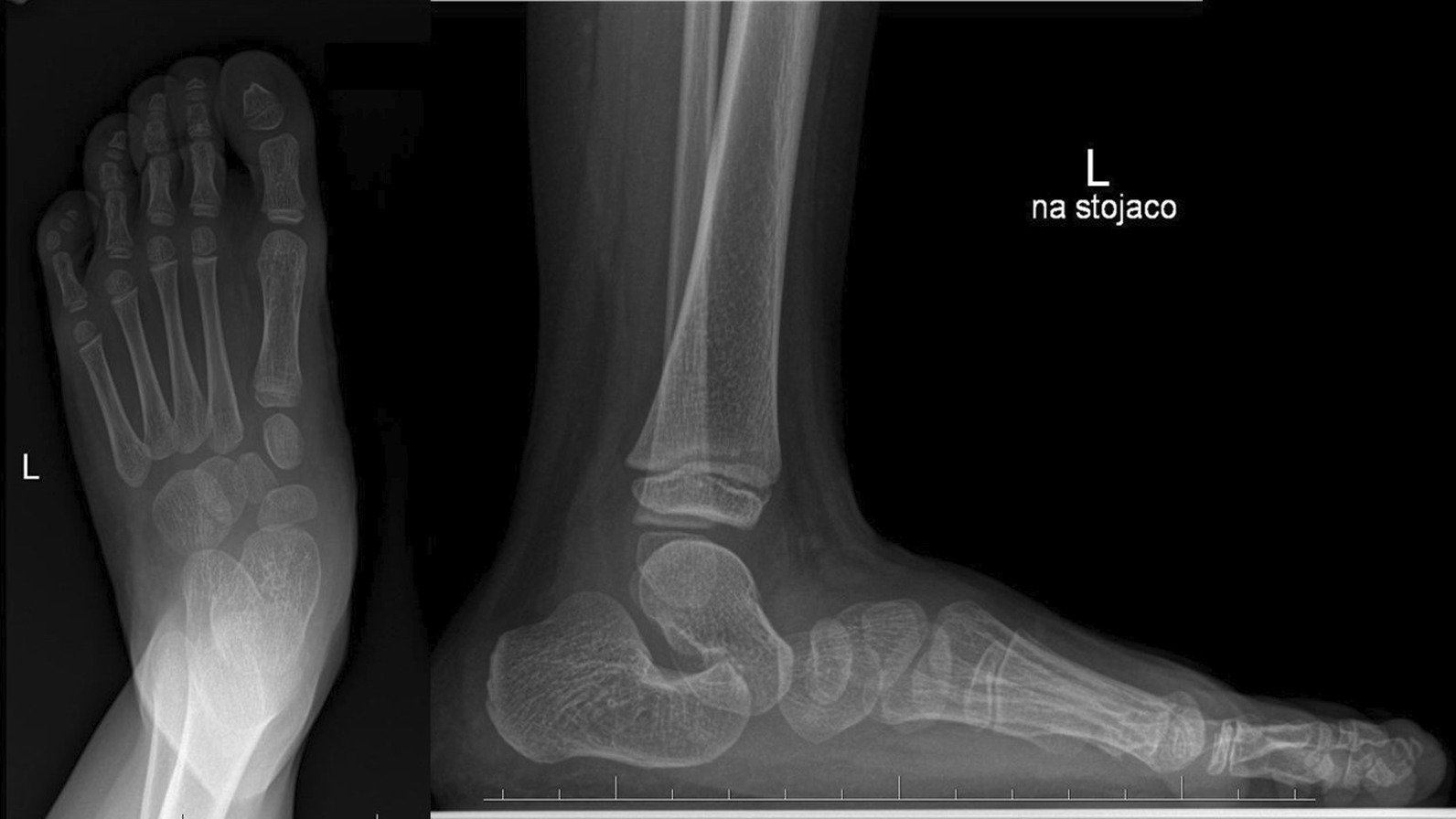
Case 1 AP and L X-ray at last follow-up. A recurrence of foot deformity is visible

**Fig. 4:**
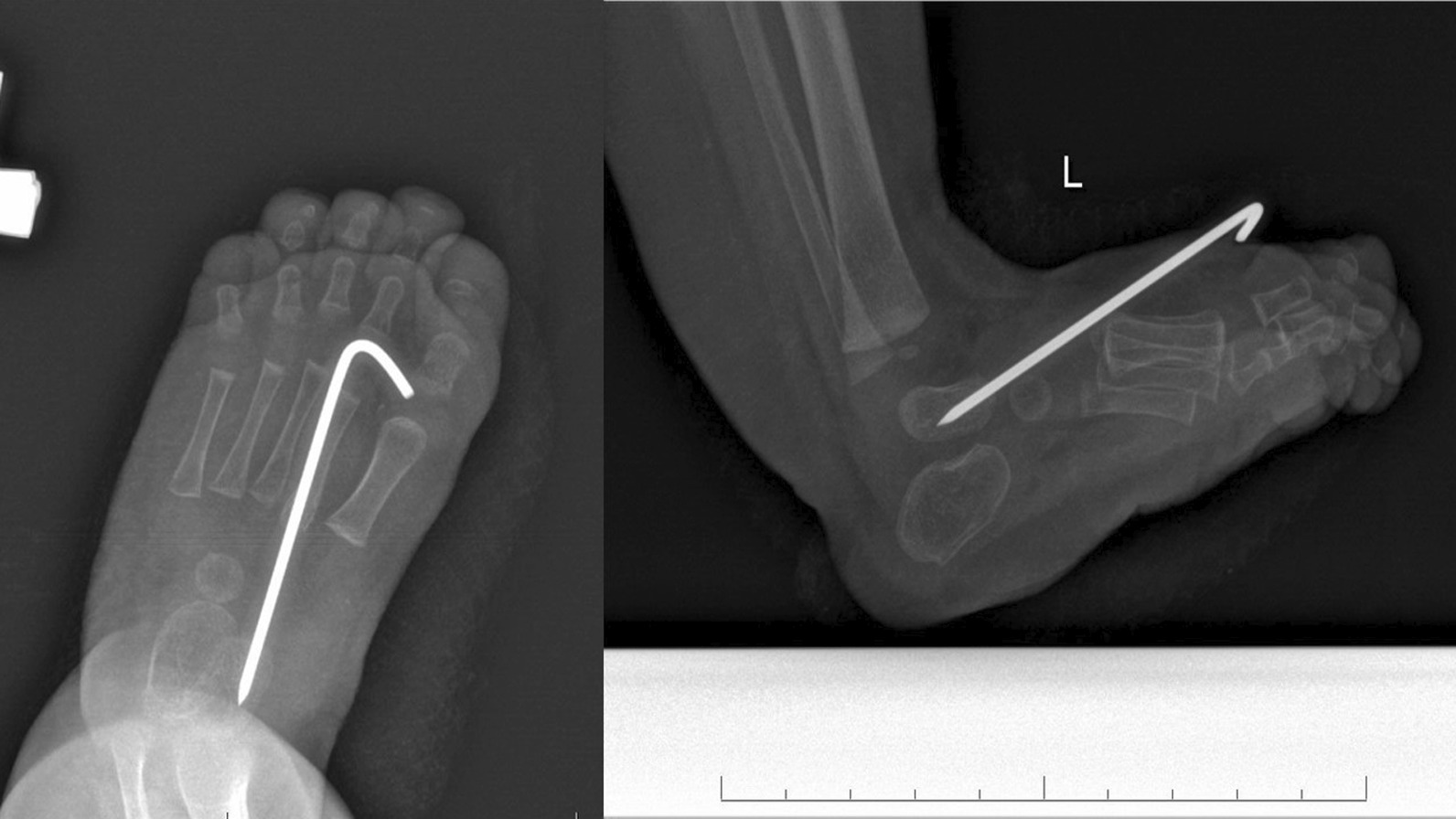
Case 1 AP and L X-ray after surgery

**Fig. 5:**
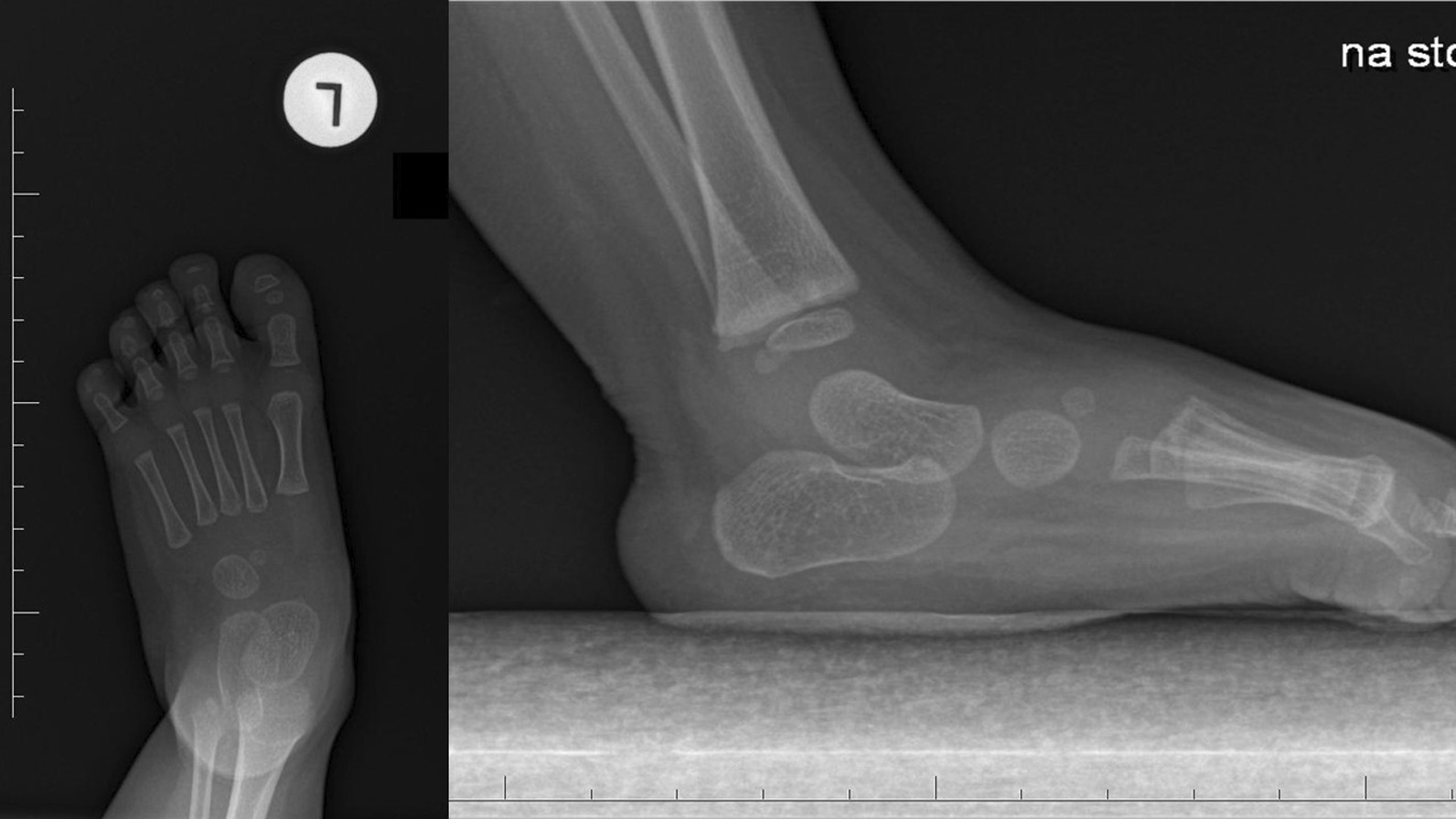
Case 1 AP and L X-ray 6 months after surgery

The final clinical assessment during the last follow-up (the mean: 35.8 months, the range: 25–52) revealed neutral position of the foot and normal range of motion in ten cases and recurrence of foot deformity in one case. The X-ray examination showed normalization all of radiological parameters at the last follow-up (Figs. 1 and 2). The mean AP talocalcaneal angle was 26.4°, lateral talocalcaneal angle—32.55°, AP talar-first metatarsal angle—18.64°, lateral talar-basis first metatarsal angle—13.64° and lateral tibiotalar angle—66.27°. We observed improper angular values in recurrence case at the last follow-up (Fig. 3). A retrospective analysis of this case showed incomplete correction from the beginning; the cuboid was dorsally subluxated at the anterior process of calcaneus at the early stage of treatment (Figs. 4, 5). Therefore it could be classified as residual deformity rather than recurrence of the initial deformity. A statistical analysis revealed that the examined X-ray parameters significantly improved during the last follow-up (Table 1).

## Discussion

**Fig. 6:**
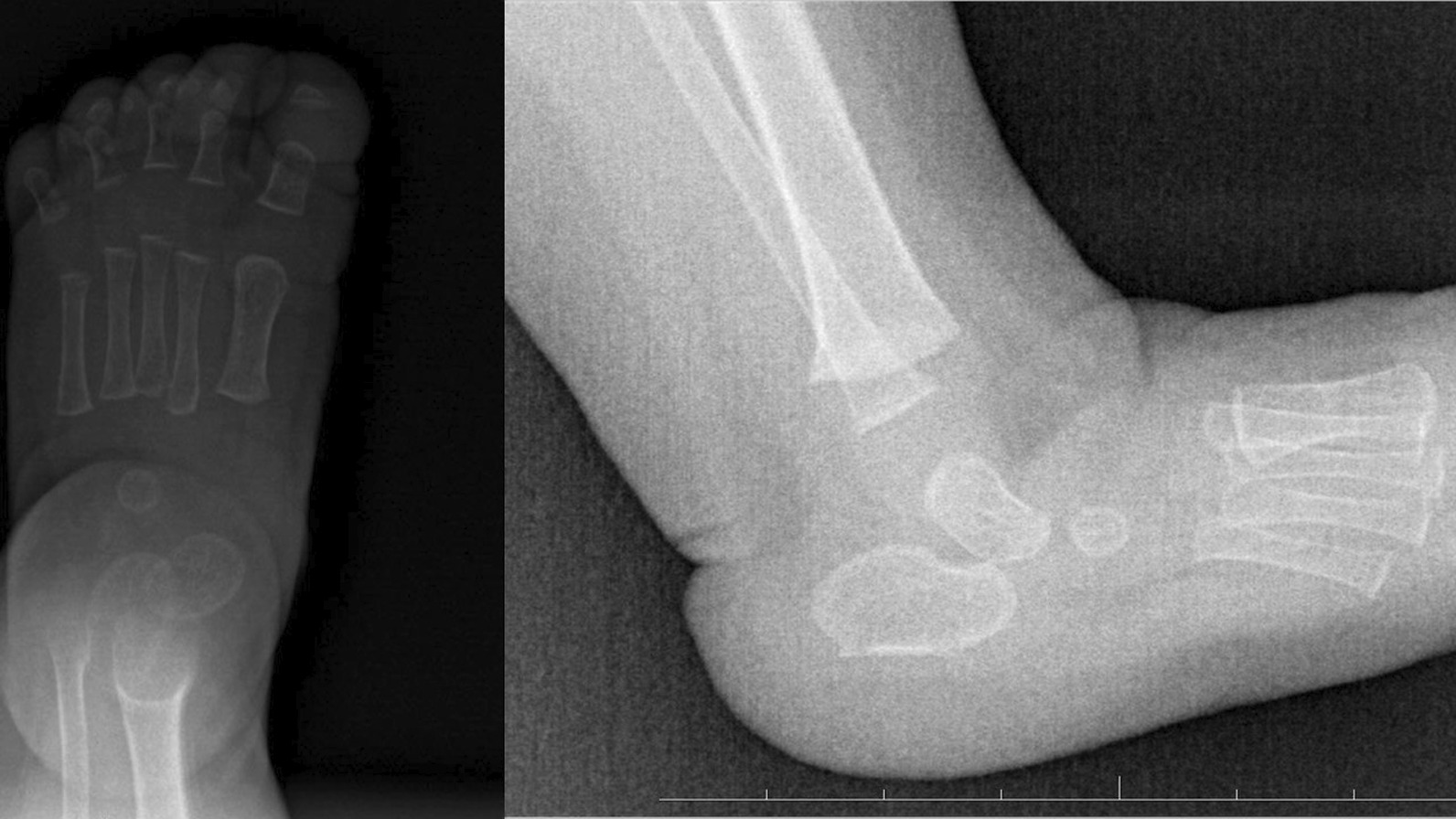
Case 1 AP and L X-ray before surgery (after 5 casts)

**Fig. 7:**
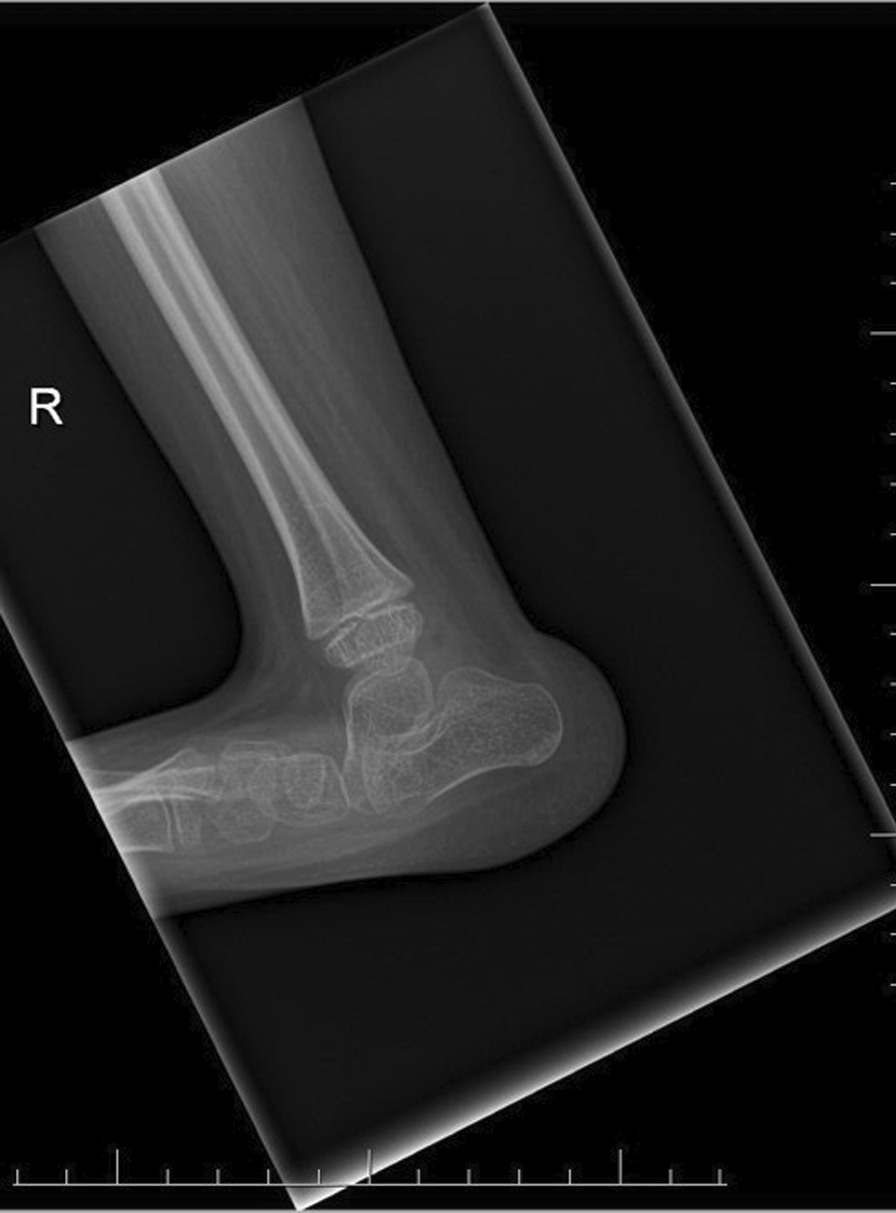
Case 2 AP and L X-ray before surgery

The exact etiology of congenital vertical talus is still undiscovered. Congenital vertical talus is a rare rigid flatfoot disorder and more than half of cases are associated with teratological disorders, which could result in a poorer final outcome [3, 7]. This foot defect is often unrecognized or misdiagnosed in infants due to a spectrum of rigidity of the foot deformity. The deformity is pretty rare and it is difficult to collect a homogeneous research group consisting of more than 8–10 cases. Hence, there are hardly few reports on vertical talus in professional literature. Radiological examinations play an absolutely essential role in diagnosis of congenital vertical talus. A weight-bearing AP and a lateral view of the foot in the maximum plantar flexion. X-ray films are required to confirm the diagnosis and assess reducibility of the deformity. A lack of ossification of many bones in the foot at birth can make the diagnosis of congenital vertical talus challenging on plain radiographs. When analyzing changes in the mutual bone arrangement in the radiological examination of the foot with suspicion of congenital vertical talus from among many evaluated radiological parameters, attention should be paid to the vertical position of the talus, the cuboid on the anterior part of the calcaneus, talocalcaneal angle and angular relationship between the talus axis and the first metatarsal bone axis (TAMBA) [6, 8, 9] (Figs. 6 and 7).

The goal of treatment of congenital vertical talus is to restore the normal anatomic relationship among talus, calcaneal and navicular bones and normal cosmetic appearance and functionality of the foot. In the past, many approaches for treatment of congenital vertical talus were both conservative and surgical [5, 10–16]. Serial casting and manipulation treatment aiming to correct congenital vertical talus has not appeared to be highly successful. Storen and Eraltug reported, respectively, 40–63.6% recurrence of deformity of the foot after serial casting [15, 16]. Since implementation of conservative treatment alone does not seem effective, surgical treatment has become the method of choice. There is a lot of controversy regarding the number and localizations of ideal approaches which could be used to surgically correct the deformity. Besides, it is still unclear whether congenital vertical talus should be corrected during one or two stages [4, 11, 17, 18]. Walker et al. proposed a 2-stage surgery, where the first stage involves lengthening contracted dorsolateral tendons, performing dorsolateral capsulotomy and reducing the talonavicular and subtalar joint complex. The second stage consists in lengthening the Achilles and peroneal tendons and performing posterolateral capsulotomy [17]. Coleman in the second stage corrected the hindfoot and the tibialis posterior tendon was advanced to the plantar aspect of the navicular [4]. The one-stage surgical approach was simply a combination of two stages [5, 11, 14, 18–20]. The surgery involved large soft tissue releases and K-wire fixation. Patients often need several intensive surgeries, such as subtalar or triple arthrodesis, in their future life. A surgery could result in some complication like wound necrosis, talar necrosis, joint stiffness and recurrence. Dobbs introduced a new approach for treatment of idiopathic congenital vertical talus in 2006. The idea includes serial manipulation and casting with the so-called reversed Ponseti technique, followed by percutaneous pinning of the talonavicular joint by using K-wire and performing percutaneous tenotomy of the Achilles tendon for the posterior contracture. For incomplete reduction in the talonavicular joint after casting he recommended an open reduction and K-wire fixation via mini incision [1].

We created a homogeneous group and collected only idiopathic congenital vertical talus in this study. All patients were treated in the same way. We used the reverse Ponseti method to achieve a correction of deformity in the talonavicular joint. K-wire fixation was always performed via mini incision regardless of the quality of the talonavicular joint correction for two reason. Firstly, a pinning was done in the retrograde way under eyes and C-arm control and secondly, the mini approach allowed for joint capsulotomy and easier bones mobilization. The results obtained in our study are comparable with those presented in the literature [8, 9, 21]. It is worth pointing that the diagnosis of congenital talus vertical is relatively late. In our material, the foot deformation was diagnosed on average at the age of 14.6 months. Such a late diagnosis makes the treatment with the reverse Ponseti method much more difficult than treatment of a club foot, which we initiate practically immediately after the baby is born. A fixed bones malalignment/shape and the contraction of joint capsules/ligaments of the foot can reduce correction of deformation. Considerable vertical talus correction with serial casting followed by a minimally open surgery have been recently reported. With a minimum follow-up of two years, patients treated by the Dobbs method demonstrate very good results regarding the correction of deformity [8, 9, 21–25]. Until the publication of Aslani’s et al., the age of 4 years was the upper time limit for correction of congenital vertical talus [9, 22, 26]. They treated patients older than 4 years (from 5 to 9 years of age) affected by both congenital vertical talus and congenital syndromes and neuromuscular condition. Dobbs method was similarly effective in the treatment of all of the mentioned patients [9].

The limitation of our study, similarly to other scientific reports on congenital vertical talus, was a small number of cases and a short follow-up period. The mentioned limitations were inevitable because of a low incidence of congenital foot deformity and used the surgical protocol was described in 2006.

The minimally invasive technique described by Dobbs should be the first option of treatment congenital vertical talus. It allows to reduce the talonavicular joint, brings good results and preserves foot mobility. The attention should be paid to early diagnosis. Longer term follow-up is indicated to determine if Dobbs technique is maintained into the end of growth.

## Data Availability

The data that support the findings of this study are available from the corresponding author, PB, upon reasonable request.
